# Malariometric Indices in the Context of Seasonal Malaria Chemoprevention in Children Aged 1.5 to 12 Years during the Period of High Malaria Transmission in the Suburban Area of Banfora, Burkina Faso

**DOI:** 10.3390/tropicalmed8090442

**Published:** 2023-09-09

**Authors:** San M. Ouattara, Daouda Ouattara, Emilie S. Badoum, Amidou Diarra, Denise Hien, Amidou Z. Ouedraogo, Issa Nébié, Alphonse Ouedraogo, Alfred B. Tiono, Sodiomon B. Sirima

**Affiliations:** Groupe de Recherche Action en Santé (GRAS), Ouagadougou 06 BP 10248, Burkina Faso; d.ouattara@gras.bf (D.O.); e.badoum@gras.bf (E.S.B.); a.diarra@gras.bf (A.D.); d.hien@gras.bf (D.H.); oz.amidou@gras.bf (A.Z.O.); i.ouedraogo@gras.bf (I.N.); a.ouedraogo@gras.bf (A.O.); a.tiono@gras.bf (A.B.T.); s.sirima@gras.bf (S.B.S.)

**Keywords:** malaria, malariometric indices, school-age children, children under five, Burkina Faso

## Abstract

Continuous monitoring of malaria epidemiology is needed in malaria-endemic settings to inform malaria control and elimination strategies. This study aimed to compare the malariometric indices between the under-fives and school-age children. We surveyed children aged 1.5 to 12 years for plasmodia carriage with the aim of including them in a longitudinal follow-up cohort. The survey took place from 7–11 September 2020 in a southwest area of Burkina Faso. Clinical and demographic data including malaria control measures were collected. A finger prick blood sample was taken for haemoglobin testing, and blood smears and dried blood spot preparation. The malariometric indices were calculated and compared between school-age children and those under the age of five. Multiple logistic regression was fitted to assess the association between malaria parasite carriage and age categories. Based on the PCR results, the parasite prevalence was 21.4% in the under-fives versus 44.2% in school-age children (*p*-value < 0.0001), with a pooled prevalence of 32.7% (CI = [28.8, 36.8]). The gametocyte prevalence was also significantly higher in school-age children (11.9%) compared to the under-fives (3.7%). Adjusted for covariates, school-age children were 2.9 times (IC = [2.0, 4.2]) more likely to carry the asexual parasite, compared to the under-fives. Malaria was moderate and stable endemic in this area and school-age children play a key role in the spread of the disease. The WHO conditional recommendation for intermittent preventive treatment of malaria in school-aged children living in malaria-endemic settings with moderate to high perennial or seasonal transmission should be implemented.

## 1. Introduction

Joint public health efforts have led to a decrease in the malaria burden in recent decades. Indeed, globally, malaria cases decreased from 241 million in 2000 to 227 million in 2019 while malaria deaths reduced from 896,000 in 2000 to 558,000 in 2019 [1]. In 2020, both malaria cases and deaths increased by, respectively, 5.8% (241 million cases) and 12% (627,000 deaths) compared with 2019; a situation attributed to health service disruptions in relation to the COVID-19 pandemic [1]. Despite the considerable decrease in its incidence, malaria remains a disease of public health interest mainly in the poorest areas of the globe. In recent years, reducing the number of global malaria cases below the symbolic level of 200 million cases seems unattainable [1]. Burkina Faso is within the top six most contributing countries to this global burden (3.4% of all global cases) [1]. In 2021, about 12 million cases of malaria were recorded in health facilities in this country, including 605,504 severe cases which led to 4355 deaths [2]. This sad observation recommends (i) strengthening of existing control measures (use of long-lasting insecticide-treated nets (LLINs), indoor residual spraying (IRS), seasonal malaria chemoprevention (SMC), and the RTS,S vaccine now), and (ii) the development of new control tools and strategies to give hope for malaria elimination and eradication. To inform malaria control and elimination, continuous monitoring of malaria epidemiology especially in the most affected countries is needed.

Children under five years are known to bear the heaviest burden of malaria in terms of cases and deaths and represent the most targeted age group in both studies and interventions [1,3,4,5,6]. However, school-age children are getting more and more attention with regard to their contribution to malaria transmission [7,8]. There is a paucity of data comparing the malariometric indices between school-age children and those targeted by the SMC, though this could help in informing the malaria control strategies.

In this study, we compared the malariometric indices between the SMC target population and school-age children in the Health District of Banfora, Burkina Faso (West Africa) in the framework of preparing a future malaria vaccine trial site.

## 2. Materials and Methods

### 2.1. Study Setting

This study was conducted in the Cascades region of Burkina Faso, precisely in the Health District of Banfora. This district is located about 465 km southwest of the capital city Ouagadougou, latitude 10°37′59″ North, longitude 4°46′00″ West, and altitude 299 m above sea level. The Cascades regional population in 2018 was estimated at 822,445 inhabitants among whom 407,073 are residents of the administrative province of the Comoé, which approximately corresponds to the Banfora Health District [9]. The climate is characterized by a rainy season from May to November and a dry season from December to April. The average rainfall is between the isohyets 1000–1200 mm per year and the average annual temperature ranges from 17 to 36 °C [9]. Malaria is the principal cause of morbidity in the Health District of Banfora and its transmission is stable throughout the year but peaking during the rainy season (June to October) [9]. *Plasmodium falciparum* accounts for more than 90% of malaria cases. The main malaria vectors are *Anopheles gambiae*, *An. arabiensis* and *An. funestus* [10]. Malaria control and prevention interventions including SMC in the under-fives, usage of long-lasting insecticide-treated nets (LLINs), and intermittent preventive treatment during pregnancy (IPTp) are being implemented in the study area [4]. The SMC targets children aged 3 to 59 months and its coverage is very high in the Health District, usually above 90% [2].

### 2.2. Study Design and Population

We analysed data obtained from a baseline malariometrics assessment aiming to screen children for inclusion in a cohort study to assess malaria morbidity with the ultimate goal of characterizing a future malaria vaccine trial site. The survey was conducted in two peri-urban areas of the Health District of Banfora (Bounouna and Nafona) from 7–11 September 2020 which corresponds to the transmission season in the study area. Included in the survey were the children of both genders, living in the study area, aged 1.5 to 12 years and whose parents or guardians have freely provided written informed consent. Children who received any antimalarial treatment within the previous 2 weeks or those with missing parasitological results were excluded from our analysis. Basic demographic data were collected namely age, gender, the use of insecticide-treated net (ITN) last night, the use of any other malaria prevention method (mosquito repellent cream or sprays, coils, or herbal or traditional preventive methods), and the parent’s literacy status. A brief clinical examination was performed and the axillary temperature was assessed using an electronic thermometer. A finger prick was performed to collect a blood sample for blood films and dried blood spot preparation as well as haemoglobin measurement.

### 2.3. Laboratory Evaluations

#### 2.3.1. Microscopic Diagnosis of Malaria

Blood smears prepared were air dried and the thin film was fixed with methanol. Dried smears were then stained with a controlled pH (7.2) Giemsa buffer solution and the malaria parasite was checked using a light microscope. The slides were examined independently by two competent readers for asexual and sexual parasite count as well as species identification. In case of discrepancy (difference of species, positive–negative readings, or ratio of the two-parasite densities > 1.5 or <0.67), a third independent reader was required, and the two most concordant results were used for reporting the final result. All the parasite species including *P. falciparum*, *P*. *malariae*, and *P*. *ovale* were assessed using microscopy.

#### 2.3.2. PCR Testing

Dried blood spots on Whatman filter papers (Whatman 3 mm, GE Healthcare, Pittsburg, PA, USA) were labelled then air dried and placed individually in a plastic bag containing a desiccant thus protecting them from humidity. DNA was extracted using the methanol method. For that, three pieces of filter paper were soaked in methanol (100 µL) for 15 min at room temperature, then the methanol was removed from the tube and the pieces were completely air dried for 1–2 h. After drying, 100 µL of sterile deionized water was added to each tube before heating them in a water bath for 15 min at 95–100 °C. During the incubation phase, the tube is vortexed every 5 min to extract the DNA. DNA extracts have been used directly. The molecular method based on DNA amplification was used to detect *P. falciparum*. This is a nested PCR method targeting the 18 s rRNA small sub-unit gene. Products obtained after the first PCR (using primers for all four *Plasmodium* species), were subsequently amplified using *P. falciparum*-specific primers. The sequence of the primers and the protocol of PCR were previously described in detail elsewhere [11]. Primary and secondary PCRs were carried out in a final volume of 20 μL containing 4 μL of Master Mix (5× FIREPol^®^, 1.25 μL of dNTP, 0.8 μL of 25 mM MgCl_2_, 0.1 μL of One Taq^®^ DNA polymerase) and 1 μL of template DNA or primary PCR. For the primary PCR, the temperature cycling parameters (24 cycles) were: initial denaturation at 95 °C for 5 min, annealing at 58 °C for 2 min, extension at 72 °C for 2 min and final extension at 72 °C for 5 min. The same program was used for the second amplification but for 30 cycles instead of 24 cycles. Samples after amplification were subjected to electrophoretic migration on a 1.5% agarose gel (Sigma Aldrich Chemie GMBH, Taufkirchen, Germany) and 3% GelRed^®^ (Nucleic Acid Stain) in Tris Borate Ethylene-Diamine-Tetra-Acetic. Migration was performed at 100 volts for 90 min. Fragment size was determined using the molecular weight marker. UV electrophoresis development was carried out using an image documentation system (AXYGEN^®^ device) coupled to a computer, enabling the exact size of DNA bands to be estimated. The expected fragment length was 205 base pairs (bp) for *P. falciparum*.

#### 2.3.3. Haemoglobin Measurement

The finger prick whole blood sample was put in a microcuvette and analysed using a portable HemoCue^®^ analyser (Ängelholm, Sweden). The result was directly read on the machine screen and immediately recorded in the case report form (CRF).

### 2.4. Statistical Methods

#### 2.4.1. Description of Variables

The parasitemia was considered positive if asexual parasites were found at microscopy whatever the parasite density. Also, to avoid missing sub-microscopic infections, we performed nested PCR. The parasite prevalence was considered as the proportion of participants with positive parasitemia. This indicator was calculated using microscopy and PCR results separately.

The specific infection prevalence was defined as the proportion of each species in the total population surveyed.

The gametocyte prevalence was defined as the proportion of participants bearing a sexual stage of malaria parasite as detected by microscopy (gametocyte counts ≥ 1 gametocyte/μL of blood), whatever the parasite species. The gametocyte prevalence was also assessed for each parasite species.

The status of anaemia was determined according to the WHO classification based on age [12]. The anaemia prevalence was the proportion of participants with a haemoglobin level less than 11 g/dL for the under-fives; haemoglobin level less than 11.5 g/dl for the 5–11 years age group; and haemoglobin level less than 12 g/dl for the 12 years old. This includes all grades of anaemia, from mild to severe. The anaemia grades (mild, moderate, and severe) were also defined based on age as indicated in the WHO classification [12].

The clinical malaria prevalence was considered as the proportion of participants who were experiencing during the survey, an episode of clinical malaria defined as measured fever (axillary temperature ≥ 37.5 °C) or history of fever within the previous 24 h plus positive blood film microscopy or positive PCR.

The main exposure variable was the exposure to SMC which depends on age groups. Two age categories were included in the study:

- Children aged 1.5 to 5 years were named the SMC target population.
- Children aged 6 to 12 years old were considered school-age children.

#### 2.4.2. Sample Size Estimation

To detect a parasite prevalence ratio of 1.5 between the two age categories, with a power of 80% and a two-sided confidence level of 95%, 254 participants per group (for equal groups) were needed (total of 508 participants), considering a non-response proportion of 10%. The overall number of participants was also enough to determine each of the malariometric indices with a confidence level of 95% and a precision of 5%.

#### 2.4.3. Data Management and Analysis

Data were directly recorded on tablets, double-checked by the investigators, and transferred to the dataset. Controls and edit checks were included in the data capture system to ensure data quality. Data were cleaned before being exported for analysis. Descriptive statistics were performed. For categorical variables, proportions and their 95% confidence interval (CI) were estimated while for continuous variables means or medians and standard deviation were evaluated. Bivariate analysis using a z-test was performed to compare the malariometric indices between age groups. The geometric mean of parasite density (GMPD) was compared between the age strata using a Wilcoxon rank-sum test.

Multiple logistic regression was fitted to assess the association between malaria parasite carriage (based on PCR results) and age categories (SMC target population vs. school-age children). Likewise, a multiple logistic regression was used to assess factors associated with gametocyte carriage. Data were analysed using Stata Version 15 and a *p*-value < 0.05 was considered statistically significant.

## 3. Results

### 3.1. Sociodemographic Characteristics of the Study Population

In total, 536 children were surveyed, 6 participants were excluded from the analysis as they were under malaria treatment using artemisinin-based combination therapies (ACTs), and no parasitological assessment was performed for them.

Out of the 530 participants included in the analysis, 269 (50.75%) were aged 1.5 years to 5 years old (considered as SMC target population) with a mean age of 3.88 years (sd = 1.25 years), and 261 (49.25%) were aged 6 years to 12 years (considered as school-age children) with a mean age of 8.93 years (sd = 1.76 years).

Both genders were equally represented in the study population with 262 males (49.43%) and 268 females (50.57%). The proportion of different genders was also comparable between age categories. In the SMC target population, the proportion of females was 50.19% while this proportion was 50.96% in the school-age children population.

Most of the study population used insecticide-treated nets (89.62%). This proportion was roughly the same between age groups, 90.71% in the SMC target population against 88.51% in the school-age children. In the study population, 83 participants (15.66%) were using other malaria prevention methods (mosquito repellent cream or sprays, coils, or herbal or traditional preventive methods) out of whom, 57 participants were simultaneously using LLINs. Majority of the children’s parents were not literate (91.51% of the total population). The demographic characteristics of the study population are presented in Table 1 below.

### 3.2. Malariometric Indices

#### 3.2.1. Parasite Prevalence

The parasite prevalence was assessed by both microscopy and PCR. The parasite prevalence in the total population using PCR data was 32.70% (172/526) (confidence interval (CI) = [28.81, 36.84]). This prevalence using microscopy results was 24.53% (130/530) (CI = [21.04, 28.38]). The proportion of sub-microscopic parasitemia (defined as parasitemia not detected by microscopy but detected by PCR) was 27.33% (47/172), IC = [21.13, 34.54]. Considering the PCR which is the most accurate test, parasite prevalence was 21.43% (57/266) in the SMC target population against 44.23% (115/260) in school-age children (*p*-value < 0.0001).

After the end of SMC (after the age of 5 years), parasite prevalence appears to be an increasing trend as shown in Table 2. Indeed, after the ending of SMC, each year of increase in age leads to a significant 26.1% increase in the odds of carrying the asexual stage of the malaria parasite (CI for the crude OR [1.17, 1.36], considering the age as a continuous explanatory variable). However, in a univariate logistic regression with a categorical age, there is no significant difference between the children under five years old, the five-year-olds, and the six-year-old children with regards to the odds of parasite carriage; this difference is instead significant for the children aged above six years (7, 8, 9, 10, 11 and 12 years old) as shown in Table 2.

#### 3.2.2. Geometric Mean of Parasite Density (GMPD)

Overall the GMPD was 815 trophozoites/µL (CI = [560, 1186]). The GMPD was 2008 trophozoites/µL (CI = [982, 4105]) in the SMC target population (32 positive participants) while it was 605 trophozoites/µL (CI = [393, 929]) in the school-age children (98 positive participants) (*p*-value = 0.0042). The GMPD was 4122 trophozoites/µL in children experiencing a clinical malaria episode (6 cases) against 753 trophozoites/µL in asymptomatic children (166 cases) (*p* < 0.0001).

#### 3.2.3. Clinical Malaria Prevalence

The clinical malaria prevalence was 1.13% (CI = [0.51, 2.50]), which corresponds to six clinical malaria episodes that were diagnosed during the survey within the study population (530 children). Most of the parasite carriers were asymptomatic (166/172 = 96.51%).

#### 3.2.4. Specific Infection Proportion

*P. falciparum* was the main parasite species. It was found in about 96% of the infection (CI = [90.99, 98.40]). This specific infection proportion was comparable between age groups. *P malariae* and *P. ovale* were found in, respectively, 6.92% (9 cases) and 0.77% (1 case) of the study population. The unique case of *P. ovale* was a mono-infection while the six cases of *P. malariae* were found in co-infection with *P. falciparum*.

#### 3.2.5. Gametocyte Prevalence

In total, the gametocyte (regardless of species) carriers as diagnosed by microscopy represented 7.74% (IC = [05.74, 10.34]) of the study population. This prevalence was significantly higher in school-age children (11.88%) compared to the SMC target population (3.72%) (*p* < 0.0001).

#### 3.2.6. Anaemia Prevalence

In this study, 48.7% (131/269) of SMC target children were anaemic, while in school-age children this proportion was 41.5% (108/260) (*p* = 0.098). Overall, 45.18% (239/529) of the children (95% CI [41.0, 49.4]) were anaemic of whom 41.4% (99/239) were mild, 57.3% (137/239) moderate, and 1.23% (3/239) severe. The interquartile range (IQR) of the haemoglobin level was [10.5, 12.3] with a median of 11.4 g/dl (95% CI [11.3, 11.6]).

The malariometric indices are summarized in Table 3 below.

### 3.3. Association between Age-Group and Asexual Malaria Parasite Carriage

The result of the logistic model showed that school-age children are more exposed to asexual malaria parasite carriage. Adjusted for sex, usage of ITNs or any other malaria prevention method, and the parent’s educational status, school-age children were 2.89 times (IC = [1.96, 4.24]) more likely to be infected with the malaria parasite (asexual), compared to the SMC target population. Table 4 presents the results of this logistic regression.

### 3.4. Factor Associated with Gametocyte Carriage

A logistic regression was also fitted to assess the factors associated with gametocyte carriage. The results indicate that the school-age children were 3.55 times (CI = [1.69, 7.47]) more at risk of carrying gametocytes compared to the SMC target population, adjusting for sex, anaemia, usage of malaria prevention methods, and parent’s educational status. Table 5 presents the results of this model.

## 4. Discussion

Malariometric indices are key indicators of malaria endemicity in an area. They should be continuously monitored as they inform on the parasite reservoir and its transmission and could give insights on the bottlenecks towards disease elimination. In Burkina Faso, the latest malaria indicator survey was conducted during the years 2017–2018 [4]. This study has provided some updates on these indicators while allowing for their comparison between the SMC target population and school-aged children.

The parasite prevalence in this study was 32.7% and was mostly due to *P. falciparum* (91–98.4%). This result indicated that malaria was a moderate and stable endemic (parasite prevalence within in the range of 10–35%) in the area [3,13]. After roughly 30 years of implementation of the national malaria control program (created in 1991), the country is still in the phase of intervention so-called “sustain” [13]. In the framework of that program, ACTs were adopted as first-line treatment in 2005, IPTp started in 2006, four rounds (2010, 2013–2014, 2016 and 2019) of free distribution of LLINs performed, and SMC started in 2014. The parasite prevalence noted by microscopy in our study (24.53% by microscopy) was about half of that noted in a previous similar study conducted in the same area during the same period in 2009 (55.2%) [5]. Like the asexual parasite prevalence, the gametocyte prevalence was also reduced by half in our study compared to this previous study [5]. In 2009 SMC was not yet implemented in the country and LLIN coverage was very low (about 32% of households had at least one net [4]) as no free nationwide net distribution was yet performed. Moreover, the study was conducted during the transmission season and children under five had received their SMC second dose one month before. These two main interventions (SMC and LLINs) could explain the low parasite prevalence found in this study. Many studies have highlighted the benefit of SMC in reducing the malaria burden in the under-five population [14,15]. However, the parasite prevalence was comparable to the prevalence (17%) reported by the last malaria indicator survey (MIS) in the country (2017–2018). As per this last MIS (carried out in the under-fives age group), the parasite prevalence in the cascade region (the study site) was 13% [4], which is similar to the prevalence found in the under-fives by microscopy in our study (11.9%).

The study found that one-quarter of the parasitemia was sub-microscopic (27.33%). This reflects the magnitude of parasite carriers not detectable by routine diagnostic tools yet contributing to the spread of the disease. Targeting sub-microscopic parasitemia with innovative interventions is a requirement for the achievement of the malaria elimination goal.

The study has shown that asexual parasite prevalence as well as gametocyte prevalence was significantly higher in school-age children compared to the SMC target population. Furthermore, adjusting for covariates (sex, usage of ITNs, usage of any other malaria prevention method, and parent literacy status) school-age children were about three times more at risk of carrying asexual parasites than SMC target children. Likewise, the odds of carrying the sexual stage of the malaria parasite were 3.55 times higher in school-age children compared to those under the age of five years. This study’s results corroborate with those of several studies assessing malaria burden in school-aged children [7,8,16,17]. For instance, in 2017 in the Cascades region of Burkina Faso, a study reported that children aged 5–10 years old and those aged 10–15 years were, respectively, 3.7 times and 3.1 times at more risk of *P. falciparum* infection compared to the under-fives [18]. In 2013 a study in Malawi reported similar findings [7]. With the implementation of the SMC, there are fears that the burden of malaria may shift from children under five to school-age children. Indeed, the SMC could delay the establishment of malaria immunity in children [17]. In addition, school-age children are less targeted by the malaria prevention intervention, use fewer ITNs, and have limited maternal care compared to the under-fives [7,17]. Moreover, the government’s policy of free health care for children under five and pregnant women in Burkina Faso [19] might have also contributed to the decrease in the malaria burden in the under-fives.

With regard to the fact that school-age children contribute widely to the malaria parasite reservoir and that this group may suffer from more uncomplicated and severe malaria cases, many authors claimed more malaria intervention towards them [7,8,16,17]. WHO also made a conditional recommendation for intermittent preventive treatment of malaria in school-aged children living in malaria-endemic settings with moderate to high perennial or seasonal transmission [3].

### Strength and Limitations

Some limits should be considered while interpreting the results of this study. The convenience sample could hamper the results’ generalisability. It has been presumed that the SMC target population has received two rounds of SMC before the survey but this was not documented and could bias the interpretation of the results. Moreover, some household characteristics like the level of sanitation and the level of wealth were not included in our analyses and this could affect the relationship between malaria indices and age groups. Nevertheless, we used PCR results for assessing the relationship between malaria infection and age group, and this helped in mitigating the misclassification bias (with regard to the parasite carriage status).

## 5. Conclusions

Despite the implementation of SMC, malaria transmission remains moderate and stable in the Health District of Banfora. School-age children are three to four times more likely to carry both the asexual and sexual stages of the parasite compared to the under-fives targeted by SMC. The positive impact of SMC in reducing the burden of malaria in children under five can therefore be inferred. The study indicated that school-age children represent a large reservoir of parasites and could significantly contribute to the spread of the disease. Further epidemiological studies are needed to determine whether this parasite carriage in school-age children leads to higher morbidity or mortality than in children under five years of age, and thus to conclude that there is a shift in malaria burden. To meet the goal of malaria elimination, effective control interventions should be implemented in school-age children.

## Figures and Tables

**Table 1:** Sociodemographic characteristics of the study population.

Characteristics	Age Groups (Years)		Total Population n (%)
	[1.5–6[ n (%)	[6–13[ n (%)	
Gender			
Male	134 (49.81)	128 (49.04)	262 (49.43)
Female	135 (50.19)	133 (50.96)	268 (50.57)
ITN usage (slept under ITN last night)			
Yes	244 (90.71)	231 (88.51)	475 (89.62)
No	25 (9.29)	30 (11.49)	55 (10.38)
Usage of any other malaria prevention method *			
Yes	43 (15.99)	40 (15.33)	83 (15.66)
No	226 (84.01)	221 (84.67)	447 (84.34)
literate parent			
Yes	25 (9.29)	20 (7.66)	45 (8.49)
No	244 (90.71)	241 (92.34)	485 (91.51)
Total	269 (50.75)	261 (49.25)	530 (100)

* Other malaria prevention methods: mosquito repellent cream or sprays, coils, or herbal or traditional preventive methods.

**Table 2:** Association between age after the stop of SMC and parasite carriage.

Positive PCR	n (%)	Crude OR [95%CI]	*p*-Value
Age Group (years)			
<5	41 (19.16)	1 (base)	
5	16 (30.77)	1.87 [0.95, 3.70]	0.070
6	15 (31.91)	1.98 [0.98, 3.99]	0.057
7	15 (34.88)	2.26 [1.11, 4.61]	0.025
8	22 (46.81)	3.71 [1.91, 7.23]	<0.0001
9	27 (58.70)	5.99 [1.91, 7.23]	<0.0001
10	13 (35.14)	2.28 [1.07, 4.87]	0.032
11	16 (55.17)	5.19 [2.32, 11.64]	<0.0001
12	7 (63.64)	7.38 [2.06, 26.42]	0.002

**Table 3:** Malariometric indices.

Malariometric Indices	[1.5–6[ (n = 269) n (%)	[6–13[ (n = 261) n (%)	*p*-Value	Overall (N = 530)	
				n (%)	95% CI (%)
Parasite Prevalence					
By PCR (*P. falciparum* only)	57 (21.43)	115 (44.23)	<0.0001	172 (32.70)	[28.81, 36.84]
By blood smear					
All Plasmodium	32 (11.90)	98 (37.55)	<0.0001	130 (24.53)	[21.04, 28.38]
*P. falciparum*	31 (11.52)	94 (36.02)		125 (23.58)	[20.16, 27.40]
*P. malariae*	1 (0.37)	8 (3.07)		9 (1.70)	[0.09, 3.23]
*P. ovale*	1 (0.37)	0 (0)		1 (0.19)	[0.02, 1.33]
Gametocyte (G.) prevalence					
All Plasmodium	10 (3.72)	31 (11.88)	<0.0001	41 (7.74)	[05.74, 10.34]
*Gametocyte falciparum*	9 (3.35)	26 (9.96)		35 (6.60)	[04.77, 09.06]
*Gametocyte malariae*	0 (0)	6 (2.30)		6 (1.13)	[0.51, 2.50]
*Gametocyte ovale*	9 (3.35)	26 (9.96)		35 (6.60)	[04.77, 09.06]
GMPD (t/µL)	2008 [982, 4105]	605 [393, 929]	0.0042	815	[560, 1186]
Anemia prevalence	131 (48.70)	108 (41.54)	0.098	239 (45.18)	[40.97, 49.45]
Mild anemia	67 (51.15)	32 (29.63)		99 (41.42)	[35.3, 47.8]
Moderate anemia	63 (48.09)	74 (68.52)		137 (57.32)	[50.93, 63.47]
Severe anemia	1 (0.76)	2 (1.85)		3 (1.26)	[0.40, 03.83]
Clinical Malaria Prevalence	3 (1.12)	3 (1.15)	0.643	6 (1.13)	[0.51, 2.50]

**Table 4:** Association between age-group and malaria parasite carriage.

Factors	Crude OR [95%CI]	Adjusted OR [95%CI]	*p*-Value
Age Group (years)			
[1.5–6[	1 (base)	1 (base)	
[6–13[	2.91 [1.98, 4.26]	2.89 [1.96, 4.24]	<0.0001
Sex			
Male	1 (base)	1 (base)	
Female	1.08 [0.75, 1.56]	1.13 [0.77–1.65]	0.532
Usage of ITNs			
No	1 (base)	1 (base)	
Yes	0.64 [0.36, 1.14]	0.48 [0.25, 0.92]	0.027
Usage of any other malaria prevention method*			
No	1 (base)	1 (base)	
Yes	0.56 [0.32, 0.97]	0.51 [0.28, 0.95]	0.033
literate parent			
No	1 (base)	1 (base)	
Yes	0.49 [0.23, 1.04]	0.54 [0.24, 1.22]	0.141

* Other malaria prevention methods: mosquito repellent cream or sprays, coils, or herbal or traditional preventive methods.

**Table 5:** Factors associated with gametocyte carriage.

Factors	Crude OR [95%CI]	Adjusted OR [95%CI]	*p*-Value
Age Group			
[1.5–6[	1 (base)	1 (base)	
[6–13[	3.49 [1.67, 7.27]	3.55 [1.69, 7.47]	0.001
Anaemia			
No	1 (base)	1 (base)	
Yes	1.79 [0.94, 3.42]	1.79 [0.92, 3.49]	0.085
Sex			
Male	1 (base)	1 (base)	
Female	0.54 [0.28, 1.04]	0.57 [0.29, 1.13]	0.109
Usage of ITNs			
No	1 (base)	1 (base)	
Yes	0.65 [0.26, 1.62]	0.53 [0.19, 1.45]	0.219
Usage of any other malaria prevention method *			
No	1 (base)	1 (base)	
Yes	0.56 [0.19, 1.62]	0.59 [0.18, 1.89]	0.372
literate parent			
No	1 (base)	1 (base)	
Yes	0.25 [0.04, 1.88]	0.35 [0.04, 2.71]	0.313

* Other malaria prevention methods: mosquito repellent cream or sprays, coils, or herbal or traditional preventive methods

## Data Availability

The dataset is available from the corresponding author upon reasonable request.

## Abbreviations

ACTs	Artemisinin-based combination therapies
EDCTP	European and Developing countries Clinical trial Partnership
GMPD	Geometric Mean of Parasite Density
IPTp	Intermittent preventive treatment in pregnancy
LLINs	Long-lasting insecticide-treated nets
MIMVaC-Africa	A Multilateral Initiative to foster the clinical development of effective Malaria Vaccine candidates in Africa
MIS	Malaria Indicator Survey
PCR	Polymerase Chain Reaction
Sd	Standard deviation
SMC	Seasonal Malaria Chemoprevention
WHO	World Health Organisation

## References

[1] World Health Organization (2021). World Malaria Report 2021. https://www.who.int/teams/global-malaria-programme/reports/world-malaria-report-2021.

[2] (2021). Burkina Faso: Ministère de la Santé. Annuaire Statistique 2020. https://www.sante.gov.bf/fileadmin/user_upload/storages/annuaire_statistique_ms_2020_signe.pdf.

[3] World Health Organization (2023). WHO Guidelines for Malaria. https://apps.who.int/iris/handle/10665/366432.

[4] Institut National de la Statistique et de la Démographie (INSD), Programme d’Appui au Développement, Sanitaire (PADS), Programme National de Lutte contre le Paludisme (PNLP) et ICF (2018). Enquête sur les Indicateurs du Paludisme (EIPBF) 2017–2018.

[5] San M.O., Alphonse O., Alfred B.T., Benjamin S., Amidou D., Issa N.O., Vaillant M., Sirima S.B. (2019). Seasonal Depiction of Malariometric Indices in Children under Five Years in a Sudanese Semi-urban Area of Burkina Faso. Int. J. Trop. Dis. Health.

[6] Emina J.B.O., Doctor H.V., Yé Y. (2021). Profiling malaria infection among under-five children in the Democratic Republic of Congo. PLoS ONE.

[7] Walldorf J.A., Cohee L.M., Coalson J.E., Bauleni A., Nkanaunena K., Kapito-Tembo A., Seydel K.B., Ali D., Mathanga D., Taylor T.E. (2015). School-Age Children Are a Reservoir of Malaria Infection in Malawi. PLoS ONE.

[8] Cohee L.M., Opondo C., Clarke S.E., Halliday K.E., Cano J., Shipper A.G., Barger-Kamate B., Djimde A., Diarra S., Dokras A. (2020). Preventive malaria treatment among school-aged children in sub-Saharan Africa: A systematic review and meta-analyses. Lancet Glob. Health.

[9] Ministère de la Santé et de l’Hygiène Publique. Direction Régionale Sanitaire Des Cascades. https://www.sante.gov.bf/detail-structure?tx_news_pi1%5Baction%5D=detail&tx_news_pi1%5Bcontroller%5D=News&tx_news_pi1%5Bnews%5D=51&cHash=2b6881ec7031613785256d89cbb00fd4.

[10] Guelbeogo W.M., Sagnon N., Liu F., Besansky N.J., Costantini C. (2014). Behavioural divergence of sympatric Anopheles funestus populations in Burkina Faso. Malar. J..

[11] Snounou G., Viriyakosol S., Zhu X.P., Jarra W., Pinheiro L., do Rosario V.E., Thaithong S., Brown K.N. (1993). High sensitivity of detection of human malaria parasites by the use of nested polymerase chain reaction. Mol. Biochem. Parasitol..

[12] WHO (2011). Haemoglobin Concentrations for the Diagnosis of Anaemia and Assessment of Severity. Vitamin and Mineral Nutrition Information System.

[13] Hay S.I., Smith D.L., Snow R.W. (2008). Measuring malaria endemicity from intense to interrupted transmission. Lancet Infect. Dis..

[14] de Cola M.A., Sawadogo B., Richardson S., Ibinaiye T., Traoré A., Compaoré C.S., Oguoma C., Oresanya O., Tougri G., Rassi C. (2022). Impact of seasonal malaria chemoprevention on prevalence of malaria infection in malaria indicator surveys in Burkina Faso and Nigeria. BMJ Glob. Health.

[15] Kirakoya-Samadoulougou F., De Brouwere V., Fokam A.F., Ouédraogo M., Yé Y. (2022). Assessing the effect of seasonal malaria chemoprevention on malaria burden among children under 5 years in Burkina Faso. Malar. J..

[16] Coalson J.E., Cohee L.M., Walldorf J.A., Bauleni A., Mathanga D.P., Taylor T.E., Wilson M.L., Laufer M.K. (2019). Challenges in Treatment for Fever among School-Age Children and Adults in Malawi. Am. J. Trop. Med. Hyg..

[17] Nankabirwa J., Brooker S.J., Clarke S.E., Fernando D., Gitonga C.W., Schellenberg D., Greenwood B. (2014). Malaria in school-age children in Africa: An increasingly important challenge. Trop. Med. Int. Health.

[18] Yaro J.B., Tiono A.B., Ouedraogo A., Lambert B., Ouedraogo Z.A., Diarra A., Traore A., Lankouande M., Soulama I., Sanou A. (2022). Risk of Plasmodium falciparum infection in south-west Burkina Faso: Potential impact of expanding eligibility for seasonal malaria chemoprevention. Sci. Rep..

[19] Matt B., Kiendrébéogo J.A., Kafando Y., Tapsoba C., Sarah, Straubinger, Metangmo P.-M. (2020). Présentation de la Politique de Gratuité au Burkina Faso.

